# Supplementary material for: A Critical View of the Application of the APEX Software (Aqueous Photochemistry of Environmentally-Occurring Xenobiotics) to Predict Photoreaction Kinetics in Surface Freshwaters
Source: Molecules. 2019 Dec 18;25(1):9. doi: 10.3390/molecules25010009 (PMC7017383; doi:10.3390/molecules25010009)
Supplement: Supplementary file 1 [file molecules-25-00009-s001.zip › 3.2.4_gcc-4.4.0.zip!/3.2.4_gcc-4.4.0/3.2.4_gcc-4.4.0/doc/octave/html/interpreter/Numeric-Data-Types.html]

Numeric Data Types - Untitled


Next: Strings,
Previous: Data Types,
Up: Top


---

## 4 Numeric Data Types

A numeric constant may be a scalar, a vector, or a matrix, and it
may contain complex values.

The simplest form of a numeric constant, a scalar, is a single number
that can be an integer, a decimal fraction, a number in scientific
(exponential) notation, or a complex number. Note that by default numeric
constants are represented within Octave in double-precision floating
point format (complex constants are stored as pairs of double-precision
floating point values). It is however possible to represent real
integers as described in Integer Data Types. Here are some
examples of real-valued numeric constants, which all have the same
value:

```
     105
     1.05e+2
     1050e-1
```

To specify complex constants, you can write an expression of the form

```
     3 + 4i
     3.0 + 4.0i
     0.3e1 + 40e-1i
```

all of which are equivalent. The letter i in the previous example
stands for the pure imaginary constant, defined as
`sqrt (-1)`.

For Octave to recognize a value as the imaginary part of a complex
constant, a space must not appear between the number and the i.
If it does, Octave will print an error message, like this:

```
     octave:13> 3 + 4 i
     
     parse error:
     
       syntax error
     
     >>> 3 + 4 i
               ^
```

You may also use j, I, or J in place of the
i above. All four forms are equivalent.

— Built-in Function: **double** (x)  
> Convert x to double precision type.
>
>
>
> **See also:** single.

— Built-in Function: **complex** (x)  
— Built-in Function: **complex** (re, im)  
> Return a complex result from real arguments. With 1 real argument x,
> return the complex result x `+ 0i`. With 2 real arguments,
> return the complex result re `+` im. `complex` can
> often be more convenient than expressions such as `a + i*b`.
> For example:
>
> ```
>           complex ([1, 2], [3, 4])
>           =>
>              1 + 3i   2 + 4i
> ```
>
>
>
>
> **See also:** real, imag, iscomplex.

- Matrices- Ranges- Single Precision Data Types- Integer Data Types- Bit Manipulations- Logical Values- Promotion and Demotion of Data Types- Predicates for Numeric Objects
